# Migrants and health in the Nordic welfare states

**DOI:** 10.1186/s40985-016-0023-6

**Published:** 2016-08-26

**Authors:** Bent Greve

**Affiliations:** University of Roskilde, Roskilde, Denmark

**Keywords:** Migrants, Welfare states, Ethnicity, Healthy paradox

## Abstract

This article probes into the health of migrants with a focus on the situation in the Nordic universal welfare states. The Nordic welfare states are further compared to each other with a comparison to the EU28 if possible, including investigation of the differences among the four Nordic countries. This is done by analyzing central parameters related to access to and inequality in health care.

The article concludes that ethnicity does not give rise by itself to differences in health care, including access to care, but can be seen as a marker of where health problems might arise due to other specific socioeconomic factors, such as the impact of economic inequality. Moreover, the healthy migrant paradox cannot be confirmed.

## Background

A high degree of similarity, active labor market policies, and a universal approach to the delivery and financing of welfare benefits and services have historically characterized the Nordic welfare states of Denmark, Norway, Sweden, and Finland. The universal approach based on legal residence in a Nordic country guarantees that migrants who legally reside in the country in the same way as other citizens have access to welfare state income transfers and welfare services based upon the specific country’s rules. This includes access to health care.

It is one thing to have formal rights to a range of services but quite another to have de facto equality in access. In principle, the Nordic health care systems formally provide equal access; however, in practice, this is not guaranteed or at least not functioning effectively. There seem to be a number of elements and aspects, such as lower income and lack of education or knowledge of the system, that result in less access therefore implying that outcome might not be equal.

This article will first briefly outline the Nordic welfare model with a focus on health, followed by methodological considerations for the analysis. Next, the knowledge of migrant’s position in society and their uses of health care including in relation to equality/inequality is discussed. It should be noted that equality in this context is understood as equality in outcome, that is, indicators such as life expectancy, admission to hospitals, and effective access to treatment. From there, examples of health care analysis in relation to migrants are given, and finally, conclusions are drawn.

The article reflects on whether the differences between native and migrants (e.g., possible impact of ethnicity) influence the degree of inequality in relation to health and access to health care. This is important for the understanding of the extent to which and the analysis of whether or not the Nordic countries can continue to be viewed as universal welfare states with a high degree of similarity. The Nordic welfare states, with Denmark as an exception, are often seen to be among the best countries to promote and ensure good health; however, increasing inequality and a weakening of the initiatives that would work to reduce it, especially in Sweden, have taken place in recent years [24].

Migrants will here be understood both as migrant workers in relation to European Union rules on freedom of movement and as people who come to a Nordic country from outside the EU as migrants, refugees, or through family reunions. This distinction is important for the right to access to Denmark, Sweden, and Finland, as the EU gives special rights to migrant workers that are not necessarily given directly to people who come from countries outside the EU, although Norway more or less applies the same rules to both. Limitations in information from different databases imply, however, that not all differences in aspects related to equality in access can be included in the analysis.

The article will also try to determine whether the so-called healthy migrant paradox—that migrants have better health than the native residents—exists in the Nordic countries [18, 30]. A possible explanation for the paradox could be that migrants are often on average younger, but there may also be unobserved confounders, including a substantial difference and diversity in the educational attainment level of migrants—from people with very high education to people who are illiterate and migrants fleeing from their home countries with issues such as post-traumatic stress symptoms. Finally, some non-Western immigrants upon arrival are eating healthier diets and consuming less alcohol than the local population but are also less physically active [12]. Thereby, their health behavior has factors pointing both to a better and worse health situation.

Meanwhile, the Nordic countries have taken different approaches to immigration, Sweden being the most liberal and Denmark the most restrictive and rigorous, with Norway’s policy falling in between [6]. This could also, in principle, be pointing towards a difference in the position of migrants related to health care and overall health in the Nordic countries.

The overall purpose of the article will be to analyze equality in health and access to health care in the Nordic universal welfare states.

## Case presentation

### Nordic welfare states and health

The Nordic countries are characterized, in analyses of welfare states, by a high degree of equality, relatively large public sector financing, relatively generous benefits, and universal access to welfare benefits and services. The Nordic health care system is also characterized by universal access, although there are relatively high user fees in various areas depending on the country (medicine, general practitioners and specialists, dentists, physiotherapists, etc.) (cf. Table 2).

The Nordic welfare states have historically been less focused on charging user fees (see also the “Examples of analysis” section), although they are now on par with most OECD countries. This may be a key parameter when looking into the possible use as this can have negative consequences for equality in access to health care [4]. There are inequities in access across all OECD countries due to user charges [7], although this is less striking in Denmark, presumably due to there being no user charge for visiting the general practitioner. Still, it can influence outcome.

There is inequality in access to health care in all welfare states—although in the health field, it is presumably smaller in the Nordic welfare states than in Bismarckian welfare states [8]. Despite criticisms of using classical welfare regime typology in the analysis of health care, the regime typology is confirmed in Bambra [1], and here, it will be used (cf. also the “A few methodological considerations” section), as a guiding element and a case both to look into whether the classical elements of the Nordic welfare states can be depicted in this more specific area and to compare the different Nordic countries against this benchmarks understanding of what a Nordic welfare state is.

Given that the Nordic welfare states often have greater equality and more universal access to services, it has often been seen as a paradox that the Nordic countries do not have the smallest inequalities in health. This is despite the fact that social epidemiology in general has argued that the more universal and generous welfare states are often better at promoting the public’s general health [2]. There may be several explanations, though this is not the focus here; see instead [3].

The Nordic welfare states generally do well with respect to the outcome of health, although this does not equally apply to Denmark, where life expectancy is lower than in many other countries in Western Europe and even in the bottom of the OECD in line with the USA [24]. Still, in the Nordic countries, a lower proportion of those aged 16+ is in bad or very bad health. This is seen as partly due to higher spending on social protection; nevertheless, Denmark has the most active response to health inequalities, and Sweden has the least compared to the other Nordic countries [11]. This is a further argument for comparing the Nordic countries, that despite being seen as belonging to the same welfare state model, there are seemingly different outcomes and different approaches to health care.

Aside from the formal access for people living legally in another country, there is also the situation for undocumented migrants. In Denmark, they have access to health care in case of need for emergency treatment. In addition to emergency care, Sweden provides full access to health services for children and care for adults that cannot be postponed. Norway has, in principle, full access for undocumented migrants, yet it is against full payment of the costs except for preventive initiatives [13]. So, here too, the position of the Nordic countries is not uniform.

### A few methodological considerations

A separate methodological problem in the analysis of migrants’ position in societies and their use of health care services is that the data tends to be unavailable because ethnic status is often not included and, in some countries registration of ethnicity, is not even possible or legally permitted. Despite more opportunities in Denmark, where the statistical registry can take a person’s country of birth into consideration [22], there are still relatively few systematic studies, and they are not often updated, cf. for a recent overview [15]. Therefore, the use of health care is seen as an indicator for health status and equality in health care outcome. Thus, it is a core problem in relation to comparative analyzes of both migrants’ health as well as health care use that many countries in Europe do not have data, and the data that is available is not necessarily of particularly high quality [25]. Also, a large comparative study argued that in relation to inequality in health for migrants, “very few papers were identified” [11].

In the analysis of migrants’ position in relation to health, an important starting point is what is meant by health inequality. It can be understood as systematic measurable differences (for example, in life expectancy, mortality). Here, it will be understood as systematic differences in health, including self-rated perceived health between different groups in society. It is clear that some differences in health are genetically determined and some are socially [3], and there are social inequalities in health not only in relation to migrants but also in relation to gender, age, education, etc.

In order to find information and knowledge in the field, databases were searched for articles using words such as migrants, health, health care, inequalities, and Nordic welfare states. Cross-references and citations were also checked in order to ensure that the most important studies were incorporated into the analysis.

Additionally, different databases from organizations (especially EU, OECD, and Danish ministries), agencies and institutions dealing with migrants were searched to find data and studies on migrants’ use of health care and their health. The OECD data used comes de facto from EU-Silc and is therefore comparable to other EU data.

Focus on especially Denmark, Sweden, Finland, and Norway, from a consideration of comparative welfare analysis, suggests that these countries form a separate cluster. Hence, the analysis should be able to inform, whether as a result of a more universalistic health care approach, if there are any special effects relative to migrants’ health conditions in these countries.

Equality will be analyzed by focusing on the use of hospitals, self-reported good health, unmet social needs, and chronic health problems, as these are good proxies for diversities within the overall health care system. Risk of poverty is used as an indicator to help explain high risk of bad health and possible difficulties in access to health care.

### Data on the use of health care in Nordic countries

This section will present a number of data related to the situation of migrants, including comparisons with the native population, followed up in the next section by some more specific studies on health and the use of health care for migrants in the Nordic countries.

One question is whether or not migrants have a different health status than ethnic natives. The overall picture is that there is a greater prevalence of mental health disorders and chronic diseases such as diabetes but less spread of cancer and heart disease. Moreover, there is less use of preventive measures, while at the same time more frequent contact with general practitioners [22]. This is in line with what Statistics Denmark has calculated in the mortality index, where the value of persons of Danish origin is set to index 100, and the figures for Western immigrants is 92 for men and 93 for women. In accordance with the theories of the healthy migrant paradox, there is, for non-Western immigrants, a significantly lower index of 80 for men and 76 for women for the period 2005–2009.^1^ However, there are significant differences depending on which country migrants come from that cannot be explained by differences in socioeconomic conditions [27].

The use of hospitals and health care can be an indicator of the migrants’ position in a society when compared with other persons’ use of the system. Table 1 shows the frequency of hospital use in 2012 in Denmark and illustrates that immigrants and descendants from non-Western countries are on average using hospitals more than persons of Danish origin. Meanwhile, the picture concerning the number of days in hospitals as regards immigrants is less clear. The higher frequency rate does not reflect differences in age, as this has been taken into consideration when making the calculations. Therefore, it indicates that some immigrants from non-Western countries who come to Denmark have a number of health problems (for example, as a result of persecution in their home country or a less healthy upbringing). It is also surprising that the rates for men who are descendants from non-Western countries have such a relatively large excess incidence both with regard to admissions and days in hospital.

**Table 1:** Comparison of the frequency of hospital use and the number of days in hospital comparing men, women, and ethnicity in Denmark in 2015 using the native population as a baseline

		Patients hospitalized	Number of days in hospital (average number per patient at hospital
Men	Total	100	100
	Persons of Danish origin	100	100
	Migrants from Western countries	79	98
	Migrants from non-Western countries	110	88
	Descendants from Western countries	96	104
	Descendants from non-Western countries	121	103
Women	Total	100	100
	Persons of Danish origin	100	101
	Migrants from Western countries	80	95
	Migrants from non-Western countries	109	85
	Descendants from Western countries	95	102
	Descendants from non-Western countries	111	98

Kilde: www.statistikbanken.dk/INDP08 fundet den 18.05.30.06.2016. Note: the index is standardized taking difference in age composition into consideration

In addition to the use of and access to welfare benefits is the question of whether the risk of living in poverty is higher or lower for immigrants. This risk is defined by a disposable income below 60 % of median income. Poverty, or risk of living in poverty, is an important indicator as one possible explanation for differences in health and access to health care services may be that more migrants live in poverty. This is due also to user charges in the universal welfare states, indicating that a part of the cost (typical especially for medicine) is restrictive for low-income groups. Although in the end, this depends on the more detailed nature of the user charge system [14]. Table 2 shows immigrants risk of living in poverty compared to other people living in each country for the year 2012.

**Table 2:** Persons in immigrant household’s risk of living in poverty compared to persons in an EU household in the Nordic countries in 2012

	Born in native household (%)	Immigrant household (%)	Ratio
Denmark	14.1	31.6	2.2
Finland	14.9	38.1	2.6
Norway	11.2	25.5	2.3
Sweden	15.4	26.8	1.7
EU28	16.3	29.6	1.8

Source: OECD(2015), settling in: OECD indicators of immigrant integration 2015

The table illustrates that even in the Nordic countries, migrants have a much higher risk of living in poverty, and thus, despite having the ambition to be countries with a high degree of equality, this is not achieved in relation to migrants. In all the Nordic countries, people living in immigrant households have accordingly a significantly higher risk of living below the EU defined limit to be at risk of poverty (e.g., 60 % of median income). It may thereby help to explain that part of health inequality is a consequence of the often poorer economic circumstances for migrants than for natives or people moving within the EU area. In the Nordic countries, compared to other welfare states, there seems to be an even higher degree of inequality with a 2.2 times greater risk of a migrant household where people are living in poverty compared to native-born households in Denmark, 2.3 in Norway, and 2.6 in Finland. In Sweden, it is 1.7 times—the only country below the EU average of 1.8. Hence, migrants in the Nordic countries seem to be more at risk than in other EU countries of living in poverty as compared to the native born population. Presumably, this can help to explain the often lower health position of migrants in the egalitarian Nordic countries.

Still, some migrants under some measures are in relatively good health. This may be explained by the fact that migration to the Nordic countries is a mixture of high-skilled individuals and young people with more limited or no education, as well as people with high rates of social and health problems already upon arrival. This is often used as an explanation for the migrant health paradox [18].

The presence and size of user fees influence the degree of similarity in health and the use of the health care system and is also a parameter for the probability of inequality in access when there are high fees. Table 3 shows the private out-of-pocket payment for health care and overall health care expenditures per inhabitant.

**Table 3:** Out-of-pocket payments share of total health expenditures and public health care expenditure per inhabitant in PPP Euros in the Nordic countries in 2012

	Out-of-pocket payments as percentages of total health expenditures	Health care expenditure per inhabitant in PPP Euros
Denmark	12.9	3209
Finland	19.6	2514
Norway	15.0 (2011)	4143
Sweden	17.5	2868

Source: Eurostat (hlth_sha_hf) accessed 30.06.2016

The Nordic countries spend more on health than the average for the EU area, Norway being the country in this comparison that spends the most money per capita and Finland the least—coming close to the EU average. The Nordic countries therefore appear to be doing better in this area than most other countries. Inversely, user fee shares are in line with what is seen in other European countries with a significant spread and variation in out-of-pocket costs. The relatively high level of user fees may help to explain why, for certain migrant groups, it can be more difficult to access the existing opportunities in health care, especially for those with an income below the poverty line.

Part of the user fees are for medicines, and it may illuminate why migrants spend less than projected on consumption of drugs, as they might not be able to afford to pay for necessary medicine.

A major difference between the Nordic countries is that there are user fees in Finland, Norway, and Sweden to visit a general practitioner, but not in Denmark [20]. The fees differ in each country, but this may still be an indication that low-income immigrants could be less likely to use doctors as a gate-keeper and thereby increase hospital use in other Nordic countries as compared to Denmark.

### Examples of analysis

This section will give examples of analysis to illustrate possible differences between persons of national ethnic origin and immigrants, including immigrants coming from inside and outside the EU if possible. As described earlier, there is a general lack of data and often great difficulty in interpreting the data that is available.

Barriers to the use of health care can be, for migrants as for others, the level of user fees, as argued above, but other barriers are language, ignorance of rights, and lack of knowledge about health and health promotion [25].

Analysis suggests that migrants from countries outside the European Union have a higher probability of depression than both second-generation immigrants, persons of national ethnic origin and immigrants from other EU countries, including when taking into account differences in the countries they live in. At the same time, they are at a much greater degree of risk of social exclusion [18]. However, the Nordic countries seem to have less social exclusion than other welfare regimes.

As stated in the introduction, there is apparently a paradox with healthier migrants, since migrants often have poorer socioeconomic conditions and may be less integrated in society. One possible explanation for the paradox is that some migrants are highly educated. A recent study related to the births of children in Sweden also seems to paint a more nuanced picture. Migrants are more likely to give birth to children with low birth weight and birth earlier, while at the same time, they have a lower risk of macrosomia and late birth [17]. Migrants in several European countries, including Denmark and Sweden, make less use of screening for breast and cervical cancer, but at the same time, as in Denmark, have more frequent contact with general practitioners [21]. They also pointed to the lack of good epidemiological data as an issue in making comparative analysis. Despite the fact that there is often contact with their family doctor, drug consumption is lower for migrants even in cases of greater disease severity, indicating that they either receive less favorable treatment or are less compliant with the advice they receive [26]. Finally, user payments for medicines have a higher impact on immigrants as they, on average, have lower incomes and less attachment to the labor market than natives.

A survey of migrants from the EU and European Economic Area showed a greater likelihood of a number of infectious diseases such as HIV, TB, and chronic hepatitis B than in the indigenous populations [30].

There are also differences between the Nordic countries. A study showed [29] that in Northern Europe, there were big differences in the risk of Turkish women having a higher rate of stillbirth or infant mortality. There was not shown to be any increased risk in Norway, a minor increase in Denmark, and a higher risk in Sweden. Thus, the Nordic countries, despite having fairly similar types of health care systems, see different outcomes, indicating that a number of other factors of socioeconomic nature can be important.

There is also variation depending on the migrants’ country of origin. A study of ethnic differences in stillbirths and infant mortality for children in Denmark during the period 1981–2003 [28], showed higher risk for Somali women than for women from Turkey and Pakistan, although overall immigrant women from the five largest groups in Denmark have an excess risk, and this risk could generally not be explained by differences in socioeconomic conditions.

Variations were shown by a study in which immigrants with Pakistani and Turkish background had an increased morbidity from heart attack for women by 132 % and for men by 74 % when compared to Danish citizens, although the figures were reduced when taking into account employment and income, indicating that at least some of the explanation of inequalities in health for immigrants can be attributed to socioeconomic conditions [23, 27].

Self-reported health is a recognized indicator of health, which shows a high degree of reliability with respect to, for example, mortality. An overview of a number of studies seems to indicate that migrants in Europe have a less well-reported level of good health even when adjusting for socioeconomic factors [19]. It also applies in Sweden, which is the only Nordic country represented in the review. There is, however, data from the EU to indicate possible differences. Table 4 shows how people born in other countries report good health compared with persons born in the respective Nordic countries and the EU28 average as a reference.

**Table 4:** Percentage of foreign and native born reporting to be in good health or better in 2012

	Native-born (%)	Foreign born (%)	Adjusted foreign born (age, income, and education) (%)
Denmark	68.5	66.1	64.4
Finland	63.5	69.4	54.9
Norway	77.3	80.1	78.0
Sweden	79.0	77.1	75.9
EU28	67.6	71.3	69.8

Source: OECD(2012), settling in: OECD indicators of immigrant integration 2015

The table illustrates a significant difference between the Nordic countries where migrants have the highest reported proportion with good health, even taking into account the difference in a number of socioeconomic factors. Denmark and Finland have an even lower level of self-reported health than the EU28 area, and this is not in line with the paradox of the healthy migrant. The Nordic countries, in this area as a whole, cannot be said to place themselves in a distinct welfare group.

The tables show how many people have medical needs that are not being met (Table 5) and the proportion with chronic health problems (Table 6).

**Table 5:** Persons reporting unmet medical needs, 2009

	Native-born (%)	Foreign born (%)	Adjusted foreign born (age, income, and education) (%)
Denmark	5.8	12.6	11.4
Sweden	12.0	16.4	16.1
Norway	2.7	3.6	3.0
	6.3	11.4	11.6
EU28	6.6	6.8	

Source: OECD(2015), settling in: OECD indicators of immigrant integration 2015

**Table 6:** Persons who do not suffer from chronic health problems in the Nordic countries and the EU28, 2012

	Native-born (%)	Foreign born (%)	Adjusted foreign born (age, income, and education) (%)
Denmark	69.0	73.4	71.3
Finland	49.4	70.5	58.9
Norway	67.6	71.4	70.6
Sweden	63.9	67.0	65.1
OECD	68.4	72.0	70.5

Source: OECD(2015), settling in: OECD indicators of immigrant integration 2015

Finland and Sweden seem to have, even after adjustment for socioeconomic conditions, a higher proportion of migrants who have medical needs that are not covered. This may be related to user fees and also to the fact that despite formally having access to health services, not all migrants choose or are able to access the system.

Table 6 paints a mixed picture where migrants do or do not suffer from chronic conditions. The situation is best in Denmark (above the EU28 average) and lowest in Finland and Sweden (when adjusted), with Norway close to average. This can be explained by the fact that the Nordic countries’ mix of labor migrants and refugees on arrival has a large number of health, including mental health, problems. These data, in contrast to that presented earlier, have a slight tendency to show the migrant health paradox.

Knowledge of initiatives targeted to migrants and their health, even in a country like Denmark, is limited [9]. However, in their study, Eskildsen et al. could assess that 2/3 of the municipalities in Denmark have a separate program for preventive health initiatives in relation to migrants. The lack of knowledge about interventions is partly due to the fact that data are often not collected on ethnicity and partly because many different actors are involved in the design of interventions, exacerbated by structural reform in 2007 of the municipalities in Denmark, when these initiatives were largely decentralized to the municipal level.

Migrants, despite the healthy migrant paradox, have a number of health problems that are not necessarily the same as those of the citizens of the country they settle in. In Denmark, a study showed that migrants generally rated their health as being worse and more often suffered from stress; see also Table 4. Diabetes is more frequent among migrants, but they are a lower risk for cancer. Their mental health is worse, particularly for asylum-seekers [16]. Migrants, not including migrant workers, have also been shown to have a higher risk of ischemic heart disease than ethnic Danes. Part of this result might be explained by the fact that the study focused particularly on refugees at higher risk—so that the migration history for the individual could play a role in relation to their health as well as their status and income [5]. Another possible explanation of the healthy migrant paradox could be that at first, just after their migration, they are in better health, but their health condition as well as the condition of their offspring worsens over time [10].

## Conclusions

Ethnic minorities in the Nordic countries have both better and worse health than ethnic resident citizens. Health thus seems not to any great extent, as might have been expected, to have a correlation with ethnicity. Rather, when ethnic groups live with poorer socioeconomic conditions, their health also deteriorates more than natives.

Ethnicity is therefore more a marker to use for prevention and outreach of social policy than a specific factor that seems to have an impact on one’s health. It does not change the fact that the welfare state’s ability to integrate different population groups in society, including ensuring greater economic and social equality, must also be able to guarantee a greater degree of parity in access to welfare services, including health care. Furthermore, it is of course necessary to examine whether there are other social and cultural factors to be aware of in order to best direct societal efforts.

Each of the Nordic countries has a different system and therefore different results, and it cannot be argued that they perform as a separate cluster of countries in all areas. There are elements that not only confirm the healthy migrants’ paradox but also indicate that migrants are not necessarily in better health, for example, a lower number self-reported as being in good health. The Nordic welfare states, thus, despite universality and generosity, also are home to migrants with poorer health than the native population and large inequality in health care usage due to, for one issue, user charges in access to health care.

Still, it seems that being a migrant in and of itself is not the sole reason for these inequalities. It is but a marker pointing to a risk factor as an individual’s socioeconomic condition seems in several areas to be a more important element preventing equality in access to health care.

## Footnotes

1
http://www.dst.dk/da/Statistik/emner/doedsfald-og-middellevetid/middellevetid.aspx
